# Efficient biodegradation of straw and persistent organic pollutants by a novel strategy using recombinant *Trichoderma reesei*

**DOI:** 10.1186/s40643-022-00581-9

**Published:** 2022-08-29

**Authors:** Ying Xia, Xinda Lin

**Affiliations:** Key Laboratory of Bioorganic Synthesis of Zhejiang Province, College of Biotechnology and Bioengineering, Zhejiang University of Technology, Hangzhou, 310014 People’s Republic of China

**Keywords:** Laccase, Solid-state fermentation, Degradation, Recombinant *Trichoderma reesei*, Lignocellulose, Cellulosic enzyme

## Abstract

Efficient biodegradation of lignocellulosic biomass needs a battery of enzymes targeting cellulose, hemicellulose, and lignin. In this study, recombinant *Trichoderma reesei* ZJ-09 with *Pycnoporus sanguineus* laccase gene was used to degrade rice straw by in situ production of laccase, xylanase, and cellulases under solid-state fermentation (SSF). Effects of parameters on key enzymes (cellulase, xylanase, and laccase) in biodegradation during SSF were investigated. Under the optimized SSF conditions, the FPA, xylanase activity, and laccase activity reached 110.47 FPU/g, 5787.59 IU/g, and 24.45 IU/g, respectively, on day 12. The obtained recombinant *T. reesei* SSF system achieved efficient degradation of rice straw with the final mass loss up to 51.16% which was 1.4-fold higher than the host strain. Further, bioconversion of rice straw into a novel laccase-enriched koji for persistent organic pollutants bioremediation (LKPB) was conducted by the optimized SSF system. LKPB was found to degrade persistent organic pollutants (POPs) effectively without the addition of mediators. 4-h removal rates of three POPs mediated by LKPB (87.21% for 2,4,5-trichlorophenol, 92.45% for nonylphenol, and 90.73% for oxytetracycline) were comparable to those achieved by laccase-co-mediator system. The newly established recombinant *T. reesei* SSF system could be potential to effectively degrade lignocellulosic wastes as well as organic pollutants.

**Graphical abstract:**

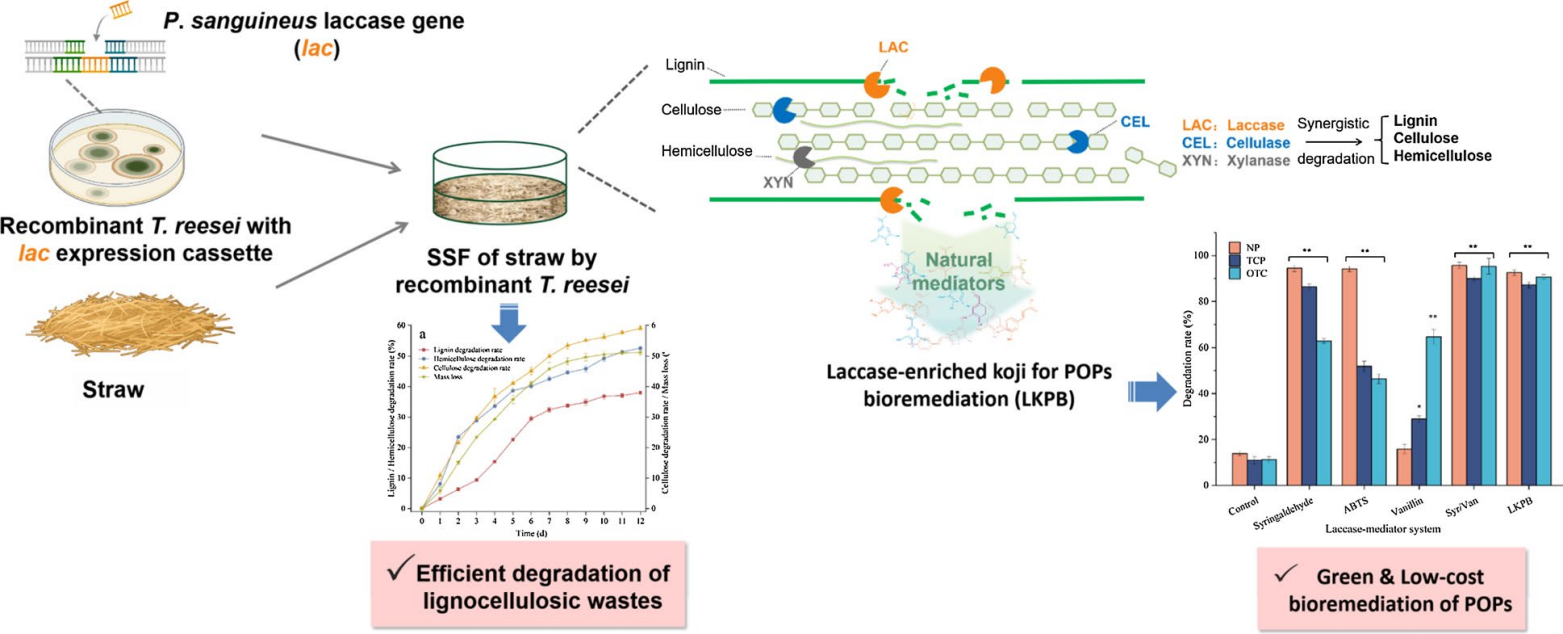

**Supplementary Information:**

The online version contains supplementary material available at 10.1186/s40643-022-00581-9.

## Introduction

Lignocellulosic biomass is one of the world’s largest low-cost raw materials for energy production. It was reported that an annual total of 700 million tons of agricultural wastes were produced in China (Sun et al. 2018; Ma et al. 2020). These wastes were derived from various types of crops such as rice, wheat, corn, and sugarcane, accounting for over 20% of the world total production (Ma et al. 2020; Ren et al. 2019).

Open-field burning is a widely used straw disposal approach in developing countries with heavy agricultural industries, such as Philippines, India, etc. Straw burning yields little exportable energy, but produces a large amount of greenhouse gases, dust, and smog, which in turn cause extensive attention of air pollution and relevant public health (Gadde et al. 2009; Pouliot et al. 2017). As statistical data have shown, about 144 million tonnes of CO_2_ and 1.4 million tonnes of PM2.5 were emitted into the air annually caused by straw burning in China (Ren et al. 2019), which suggests that conventional straw treatment based on burning is confronting increasing challenges in consideration of its environmental sustainability.

As a traditional strategy, returning straw to fields has been applied for decades and is considered an economical strategy to minimize soil erosion and water losses by improving soil structure, soil fertility, soil organic matter, and microbial activity. According to previous studies, the crop yield represented an increase of 5–10% after returning straw to the field (Ren et al. 2019). However, the low degradation rate of straw has been considered a committed step in large-scale implementations considering the fact that straw contains a large proportion of poorly biodegradable lignocellulose polymers (i.e., cellulose, hemicellulose, and lignin).

Biodegradation is a well-known environmental-friendly method which has the benefit of uncomplicated equipment, low energy cost, inhibitors production or free of chemicals, and easy implementation compared to traditional chemical and physical pretreatment approaches. The current biological pretreatment method consists mainly of enzymatic saccharification and microbial degradation mediated by bacteria or fungi (Sánchez 2009). Previous research efforts have shown that there are multiple fungi that can degrade straw such as white-rot (i.e., *Phanerochaete chrysosporium* and *Trametes versicolor*), brown-rot (i.e., *Postia placenta* and *Gloeophyllum trabeum*) (Ray et al. 2010), and soft-rot fungi (i.e., *Trichoderma reesei* and *Aspergillus niger*) (Schilling et al. 2012; Shirkavand et al. 2016). For example, applying white-rot fungi *Trametes versicolor* can improve the hydrolysis of wheat straw with 91% sugar yield improvement compared to untreated straw. However, this method has two drawbacks. First, the whole process needs to take at least 15 days to complete. Second, although white-rot fungi pretreatment removes lignin efficiently, with high selectivity for lignin degradation over cellulose loss (Wan and Li 2012), they are generally poor in removing cellulose and hemicellulose (Ma et al. 2020). Therefore, some other researches focus on using bacteria for biodegradation, including *Clostridium* sp. (Lin et al. 2011), *Cellulomonas* sp. (Sharma et al. 2019), *Bacillus* sp. (Maas et al. 2008), *Rhodococcusjostii* sp., and *Sphingobium* sp. (Xu et al. 2018), various bacterial species have been developed for lignocellulosic materials degradation. Compared to fungi, bacteria can quickly decompose cellulose and hemicellulose while they have lower efficiency in degrading lignin (Ma et al. 2020). Therefore, strategies focusing on synergistically degrading all three major components of straw should be promising in future straw management.

Laccase (EC 1.10.3.2) is a part of the family of multicopper oxidases which catalyze the one-electron oxidation of multiple phenolic molecules to phenoxy radicals accompanied by the reduction of molecular oxygen to H_2_O (Su et al. 2018). Laccases are capable of directly oxidizing the phenolic subunits (accounting for 30% of lignin) with polymeric lignin as substrate (Hilgers et al. 2018). However, the non-phenolic units (accounting for 70% of lignin) possess redox potentials up to 1500 mV and thus cannot be directly oxidized via laccase-mediated reactions (Munk et al. 2015). Nevertheless, with the existence of mediators which are small aromatic molecules acting as “electron shuttles”, laccases can act on non-phenolic parts of lignin (Christopher et al. 2014). In laccase–mediator systems (LMS), the laccase first oxidizes the mediator, which subsequently attacks non-phenolic units. In this way, the substrate range of laccases can be largely extended (Hilgers et al. 2020; Munk et al. 2015).

Besides lignin degradation, the fact that laccases only require molecular oxygen for catalysis makes them suitable for bioremediation of xenobiotic compounds (Baldrian 2006). The capability of laccase to efficiently detoxify various persistent organic pollutants (POPs) has gained increasing attention in the area of bioremediation. The contaminants investigated include dyes (Liu S et al. 2020), polycyclic aromatic hydrocarbons (PAHs) (Zeng et al. 2021), endocrine disrupters (Bayramoglu et al. 2018), and pesticides (Sarker et al. 2021).

*Pycnoporus sanguineus* isolated from the tropical area could produce the laccase with good thermostability (Garcia et al. 2007). However, this laccase reveals low enzyme activity and stability under industrial conditions, thereby limiting its applications (Agrawal et al. 2018). *Trichoderma reesei*, encoding a series of cellulases and hemicellulases, is one of the most extensively studied cellulolytic microorganisms for biomass degradation. As the major industrial cellulase producer, the engineered *T. reesei* has been reported to achieve an extracellular protein titer up to 80.6 g/L under industrial fermentation conditions (Fonseca et al. 2020). Two main methods of *T. reesei* cellulase production are submerged fermentation and solid-state fermentation (SSF). SSF is widely used as it is easy to operate, has no need for skilled manpower, and is less capital intensive since it enables the usage of cheap lignocellulosic feedstock as substrates for enzyme production (Manan and Webb 2020).

In our previous work, the laccase gene from *Pycnoporus sanguineus* was cloned and integrated into the chromosomes of *T. reesei* ZU-02 and constructed the recombinant *T. reesei* ZJ-09 (Zhao et al. 2018). The principal goals of this study were: (1) to provide more efficient recommendations for agricultural crop residues management using recombinant *T. reesei* under suitable solid-state fermentation conditions; (2) to develop novel POPs biodegrading koji via SSF by the recombinant *T. reesei*. This research will provide useful information about improving the speed of agricultural wastes and POPs biodegradation.

## Material and methods

### Strain and chemicals

*Trichoderma reesei* ZJ-09 with *Pycnoporus sanguineus* laccase gene used for the present study was constructed in our previous study (Zhao et al. 2018). The parental strain *Trichoderma reesei* ZU-02 (originally derived from ATCC56764 via rounds of screening focused on high cellulase production) was used as a control for enzyme production, biodegradation of rice straw, and POPs degradation experiments. For inoculum preparation, 3–5 mL of sterile saline 0.9 wt. % was added into fully sporulated slants and then was gently pipetted up and down. After that, the obtained spore suspension was adjusted to 1 × 10^8^ spores/mL using sterile saline.

POPs (2,4,5-trichlorophenol, nonylphenol, and oxytetracycline) were purchased from Sangon Biotech (Shanghai, China). Syringaldehyde, vanillin and 2,2-azino-bis (3-ethylbenzothiazoline-6-sulfonic acid) (ABTS) were obtained from Sigma-Aldrich (Saint-Louis, MO, USA). Methanol, oxalic acid, and acetonitrile were of HPLC grade and obtained from Sigma-Aldrich. All other chemicals used in this study were of analytical grade.

### Raw material, pre-processing and composition analyses

Wheat straw (WS), corn straw (CS), rice straw (RS), and sugar cane bagasse (SCB) were picked up from the surrounding areas in Hangzhou, Zhejiang province, China. The samples were washed thoroughly with water and air-dried. After that, samples were milled to a particle size between 2—5 mm and stored in air-tight containers until used.

Milled samples were saturated with ammonium hydroxide (solid-to-water ratio of 1:10 (w/v)) for 24 h as described previously (Chen et al. 2009). After pretreatment, the samples were washed with water until the pH reached neutral and then air-dried.

Compositional analyses of agricultural residues (pretreated and untreated) were performed according to the National Renewable Energy Laboratory (NREL) protocol (Sluiter et al. 2004). In short, two-step acid hydrolysis with 72% H_2_SO_4_ was used to fractionate the biomass into forms that were more easily quantified. The acid-insoluble material was measured by gravimetric analysis. The acid-soluble lignin was determined by UV–Vis spectroscopy. The polymeric carbohydrates were hydrolyzed into the soluble monomeric forms, which were measured by HPLC (Waters) with an Aminex HPX-42A column (Bio-Rad, USA). Hemicellulose was measured as the sum of xylose, galactose, mannose, and arabinose. Cellulose was determined as the total amount of glucose.

### Straw degradation under solid-state fermentation

Unless otherwise illustrated in the paper, solid-state fermentation by recombinant *T. *reesei was performed in 500 g capacity trays using rice straw as substrate with relative humidity maintained at 70%. 80 g of a total dry substrate and 20 g bran were mixed with 250 mL SSF nutrient solution. *T. reesei* spore suspension containing ~ 1 × 10^8^ spores/mL was inoculated into the tray with the inoculum ratio of 10% (v/w). Fermentations were carried out at 30 ℃ for 10 days. The solid-state medium was sampled each day for determinations of enzyme activities and straw degradation rates.

SSF nutrient solution containing 2% (NH_4_)_2_SO_4_, 0.5% urea, 0.5% CaCl_2_, 0.5% KH_2_PO_4_, 0.25% MgSO_4_·7H_2_O, 0.05% CoCl_2_, 0.025% CuSO_4_. The pH was adjusted to 5.0 using 50 mM citrate buffer.

### Enzyme preparation

Koji obtained from recombinant *T. reesei* ZJ-09 SSF for 10 days (LKPB) was taken directly for POPs degradation.

Enzyme extraction of LKPB was performed using 50 mM citrate buffer (pH 4.8) with a solid-to-liquid ratio of 1:5. The mixture was shaken for 60 min at 30 ℃, 200 rpm. After that, the mixture was centrifuged for 5 min at 4 ℃, 10,000 rpm. The supernatant was taken for enzyme assay.

Enzyme preparation from submerged fermentation was according to previously described (Xia et al. 2018). 1 mL of spore suspension was inoculated into 50 mL of the seed medium and grew for 48 h at 30 ℃, 180 rpm. Then 2.5 mL of the seed culture was transferred to 50 mL of the fermentation medium. Cultures were incubated at 28 ℃ up to 120 h. After that 48 h of cultivation, 2% (w/v) lactose was added to the medium every 24 h. The supernatant of the 120 h culture with laccase activity of 8.9 IU/mL was used as a control in the organic pollutants degradation experiment.

### Organic pollutants degradation

50 mg/L of 2,4,5-trichlorophenol (TCP), nonylphenol (NP), and oxytetracycline (OTC) were incubated separately with 0.2 IU/mL laccase obtained by submerged fermentation or solid-state fermentation (shown in “Enzyme preparation” section) in the presence or absence of 0.3 mM individual mediator (syringaldehyde or vanillin or ABTS) or syringaldehyde/vanillin (Syr/Van) complex at the syringaldehyde to vanillin with a ratio of 4:6. Degradation reactions were performed at pH 4.0, 50 ℃ in a shaking water bath at 120 rpm for 4 h. A control test was performed on 50 mg/L individual pollutant with 0.2 IU/mL laccase obtained by submerged fermentation without mediators.

Residual pollutant concentrations were determined by HPLC system (shown in “HPLC analysis” section).

## Analysis

### Enzyme assays

Total cellulase activity (filter paper activity, FPA) was analyzed based on the Laboratory Analytical Procedure published by NREL (Adney and Baker 2008). One FPU was defined as the amount of enzyme that produced 1 μmol of reducing sugar per minute in glucose equivalents.

Laccase activity was measured as previously reported using ABTS as the substrate (Zhao et al. 2018). One unit of laccase activity (IU) was defined as the amount of enzyme that oxidized 1 μmol of ABTS per minute.

Suspension of D-xylan 1% (w/v) in 50 mM sodium acetate buffer (pH 5.0) was used as a substrate for xylanase activity assay. 0.5 mL of diluted enzyme solution was added and the mixture was incubated at 50 ℃ for 30 min. DNS solution was used to stop the reaction. One unit of xylanase activity (IU) is defined as the amount of enzyme releasing 1 μmol of reducing sugars per minute.

β-Glucosidase activity (BGA) (Xia et al. 2018), cellobiohydrolase activity (CBHA) (Fang and Xia 2015), and endoglucanase activity (CMCase) (Miller 1959) were assayed as previously reported. One unit (IU) of BGA and CMCase was defined as the amount of enzyme that released 1 µmol glucose equivalent per minute. One unit (U) of CBHA was defined as the amount of enzyme required for generating 1 mg of reducing sugars in 1 h.

For each enzyme assay, the mean value of three independent replicates equals the absolute enzyme activity (referred to as enzyme activity). For calculation of the relative enzyme activity in SSF optimization, enzyme activity from recombinant *T. reesei* ZJ-09 through solid-state fermentation using rice straw as substrate plus 30% bran content at 30 °C, initial pH 5.0, 70% water content with 10% (v/w) initial inoculum was set to 100%. The quotient of the absolute enzyme activity and the highest value equaled the relative enzyme activity. All results were given as the mean value ± standard deviation.

### HPLC analysis

An HPLC (Agilent 1200, USA) system equipped with an Eclipse XDB-C18 column (150 mm × 4.6 mm × 5 μm) and a photodiode array detector (220 nm for TCP, 277 nm for NP, and 355 nm for OTC) was used.

Add 5 mL of methanol to 5 mL reaction system to completely dissolve the pollutants. After that, 1 mL of the sample was filtered through a 0.45-μm sterile membrane. For TCP and NP, the mobile phase was composed of methanol and water (85:15, v/v). For OTC, the mobile phase was composed of 0.01 M oxalic acid, acetonitrile, and methanol (72:14:14, v/v/v). The column was set at 30 ℃ and the injection volume was 20 μL. Flow rate was set at 1.0 mL/min for NP and TCP, and 0.8 mL/min for OTC:

$$ {\text{Degradation}}\,{\text{rate}}\,\left( \% \right) = \left( {{\text{C}}_{0} - {\text{C}}_{1} } \right)/{\text{C}}_{0} \times 100, $$

where C_0_ (mg/mL) was the initial concentration of pollutant and C_1_ (mg/mL) was the residual concentration of pollutant.

### Statistical analysis

All data in this paper are presented as the mean value ± standard deviation (SD). SPSS 20.0 (SPSS Inc., Chicago, IL, USA) was employed for statistical analysis. One-way ANOVA or t-test was used for significance of differences analysis. *p* < 0.05 was considered significant, *p* < 0.01 was considered extremely significant.

## Results and discussion

### Optimization of solid-state fermentation with recombinant *T. reesei*

Lignocellulolytic enzymes are essential to the complete decomposition of lignocellulosic biomass. Therefore, in this part, culture conditions for the yield of cellulase, laccase, and xylanase produced by recombinant *T. reesei* ZJ-09 during solid-state fermentation of lignocellulosic wastes were investigated using one-factor-at-a-time (OFAT) experiments.

### Crop type

During SSF, solid substrate is critical to microbial growth and enzyme activity, since it not only supplies the culture nutrients, but also serves as an anchor for the microbial cells (Wong et al. 2017). In this study, enzyme production by *T. reesei* ZJ-09 under SSF was evaluated using several types of agricultural wastes (wheat straw (WS), rice straw (RS), corn straw (CS), and sugar cane bagasse (SCB)) as carbon sources.

**Fig. 1:**
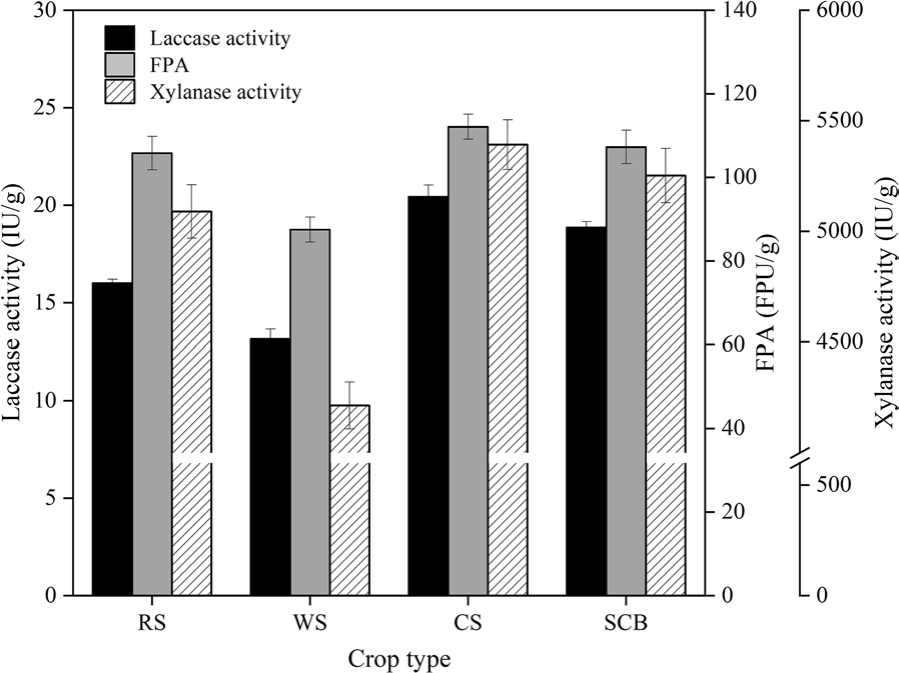
Effect of crop type on enzyme production by *T. reesei* ZJ-09 under SSF. All samples are collected on day 10 to assay the FPA, xylanase activity, and laccase activity. Data were average values of triplicate samples and error bars indicate standard deviations

Among these carbon sources, CS could induce the highest total cellulase activity (FPA) of 112.26 FPU/g, laccase activity of 20.50 IU/g, and xylanase activity of 5401.23 IU/g, respectively (Fig. 1). An explanation for this result is that CS had higher cellulose content compared to other lignocellulosic wastes (Additional file 1: Table S2) which led to the upregulation of genes associated with cellulase, hemicellulase, and laccase synthesis given that cellulose is a strong inducer for gene expression in *Trichoderma reesei* (Bischof et al. 2013). Besides CS, recombinant *T. reesei* also exhibited strong capability of cellulase, xylanase, and laccase production using RS and SCB as the substrate, indicating *T. reesei* ZJ-09 has great potential for degradation of various agricultural residues.

Rice straw, an economically feasible carbon source commonly used as the substrate for SSF, is the most abundant lignocellulosic biomass in southern China (Abraham et al. 2016). It is rich in several nutrients, which could explain its better performance as it can enhance cell growth as well as metabolism. Considering the nature of the substrate, cost, and availability, rice straw was chosen as the carbon source for further experiments.

### Bran content

It has been reported that the addition of wheat bran into SSF medium could provide adequate essential nutrients as well as inducers for microbial growth and enzyme secretion (Farinas 2015). Meanwhile, bran was discovered to have suitable particle sizes, good porosity, and offer fungi the anchorage to grow on during SSF process (Sun et al. 2008). Besides, its texture was kept loose even in moist conditions, thus providing a large surface area by holding water (Kar et al. 2013).

**Fig. 2:**
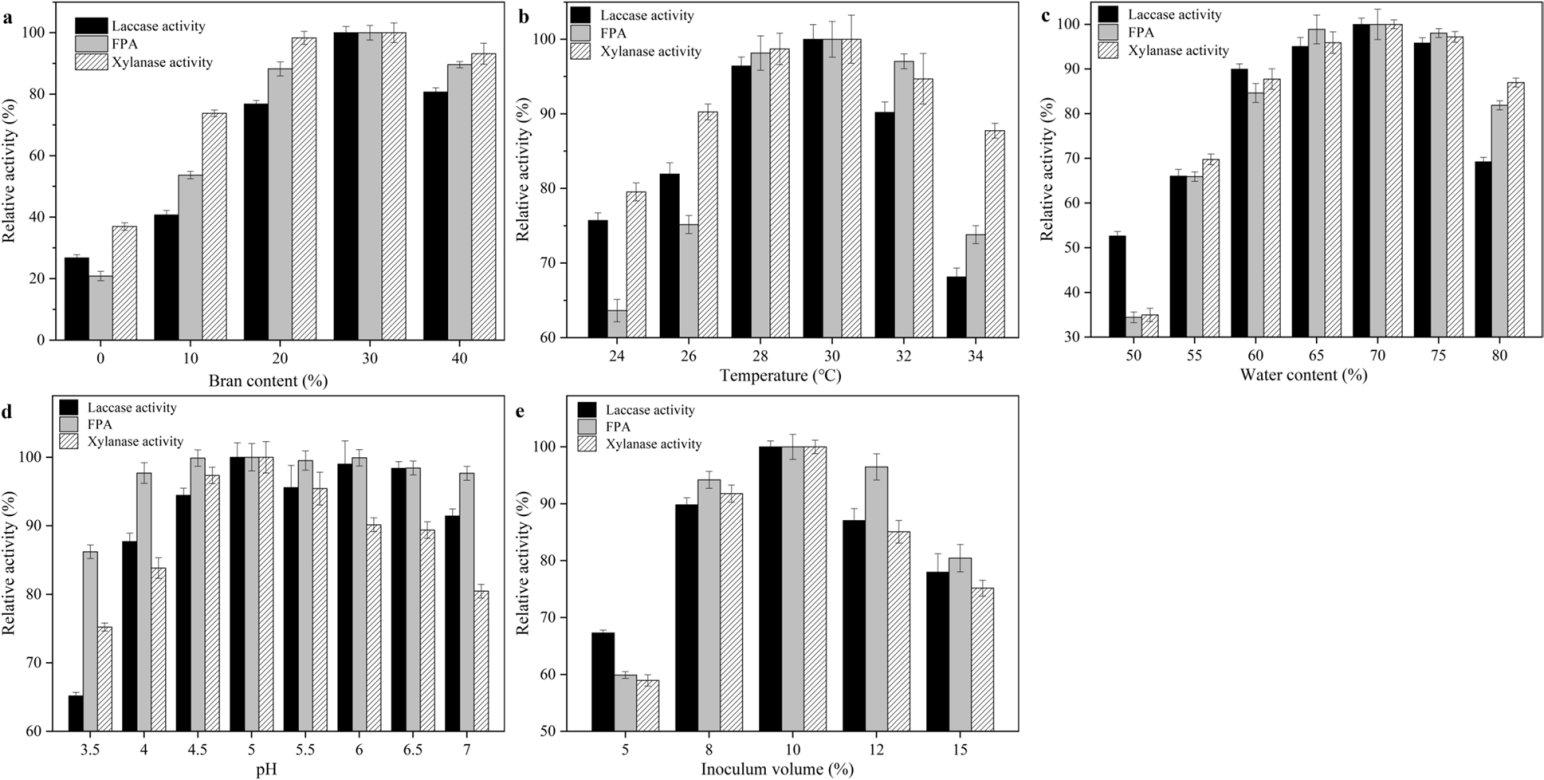
Optimization of fermentation conditions, including the effect of bran content (**a**), temperature (**b**), water content (**c**), pH (**d**), and inoculum size (**e**) on enzyme production by *T. reesei* ZJ-09 under SSF. All samples are collected on day 10 to assay the FPA, xylanase activity, and laccase activity. Data were average values of triplicate samples and error bars indicate standard deviations

In this study, the optimal bran content for enzyme production from recombinant *T. reesei* through solid-state fermentation using rice straw as substrate was investigated at 30 °C, initial pH 5.0, 70% water content with 10% (v/w) initial inoculum over a bran content from 0 to 40%. As presented in Fig. 2a, FPA, laccase activity, and xylanase activity increased with bran content, and 30% bran content led to the maximum enzyme activity. After that, further bran content increase brought slight decreases in enzyme activities.

Wheat bran is a cheap carbon source rich in several nutrients including cellulose, hemicellulose, protein, and essential minerals. It can also increase the surface area of mixed substrates, which may provide optimum support for enzymes production and cell growth of *T. reesei* ZJ-09.

### Temperature

Temperature is an important indicator during SSF, which can affect enzyme production efficiency. The effect of temperature on enzyme production during SSF was evaluated from 24 °C to 34 °C. As presented in Fig. 2b, enzyme activities did not exhibit huge differences during the measured temperature range. Three enzyme activities reached maximum values when the temperature was set to 30 °C. A slight decrease in laccase activity occurred at temperatures higher than 32 °C. Laccase activity exhibited a sharp decrease (accounting for only 68.15% of the maximal activity) when the temperature was up to 34 °C.

### Water content

Sufficient moisture is crucial to microbial growth and metabolism. It is previously reported that initial water content had a major impact on the cellulases production. In this study, different moisture contents (50—80%) were evaluated for cultures of *T. reesei* ZJ-09 under SSF using rice straw as substrate. As presented in Fig. 2c, the ideal water content was observed between 60 and 75%. FPA, laccase activity, and xylanase activity maintained above 80% of the highest enzymatic activities when moisture content varied in this range, indicating recombinant *T. reesei* ZJ-09 could thrive on a comparatively wide range of water content. 70% was proven to be optimal for enzyme production. When moisture was maintained at 70%, FPA, laccase activity, and xylanase activity reached 124.49 IU/g, 24.60 IU/g, and 5763.34 IU/g, respectively (data not shown). Further increase in moisture level had a negative impact on enzyme production.

### pH

The optimal pH for enzyme production by *T. reesei* ZJ-09 under SSF was studied over a pH range of 3.5–7.0. The FPA, laccase activity, and xylanase activity were measured on the 10th day of fermentation. Enzyme activities from the recombinant *T. reesei* did not vary considerably at pH varying from 4.5 to 7.0 (Fig. 2d). Notably, though the laccase activity decreased when pH was lower than 4.0, the recombinant *T. reesei* still presented laccase production capacity, suggesting comparatively wide pH adaptability of *T. reesei* ZJ-09. Taken together, the pH of SSF for *T. reesei* ZJ-09 was set at 7.0 (natural pH) to simplify pH adjustment process.

### Inoculum size

The effect of inoculum size on *T. reesei* SSF was studied over the range of 5–15%. As shown in Fig. 2e, the increase in inoculum size within the range of 5% to 10% resulted in increased enzyme activities which could be due to enhanced growth rates achieved in the initial phase. There is a trade-off between strain growth and enzyme production, if increase the inoculum size, the fungal growth rate can be accelerated, but at the same time nutrient depletion can be also aggravated. Fungal growth will be affected via nutrient depletion, which would do harm to improving the yield of enzymes, thereby the suitable inoculum size was set to 10% in this study.

### Enzyme production by recombinant *T. reesei* on rice straw

**Fig. 3:**
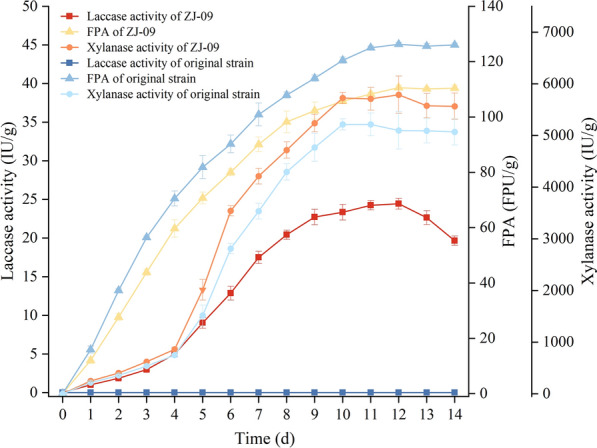
Cellulase, xylanase, and laccase production by recombinant *T. reesei* ZJ-09 and original strain under optimized SSF conditions using rice straw as substrate. Error bars represent the standard deviation of three independent repeats

The changes of enzyme activity of cellulase, laccase, and xylanase during the SSF process are given in Fig. 3. Similar to the original strain, recombinant *T. reesei* ZJ-09 had strong cellulase and xylanase producing capability. FPAs of *T. reesei* ZJ-09 and the host strain *T. reesei* ZU-02 increased rapidly from day 2 to day 7, peaking at 110.47 FPU/g and 126.27 FPU/g, respectively, on day 12. Taking other components of cellulase complex into consideration, ZJ-09 exhibited slightly lower β-glucosidase activity, cellobiohydrolase activity, and endoglucanase activity (Additional file 1: Table S1). Significant increases in xylanase activities were found after 4 days of fermentation, reaching 5723.39 IU/g and 5225.21 IU/g, respectively, on day 10.

Notably, compared to the host strain, *T. reesei* transformant ZJ-09 could persistently secrete laccase from day 4 to day 10 with the activity of 24.45 IU/g on the 10th day of fermentation.

Taken together, recombinant *T. reesei* kept the abilities of efficient production of cellulase and xylanase from the original strain. Meanwhile, *T. reesei* ZJ-09 could use rice straw as a substrate to produce high activity laccase.

It was known that the complete degradation of lignocellulosic biomass needs a battery of enzymes targeting cellulose, hemicellulose, and lignin. Because of its complexity and rigid structure, lignin is hard to degrade. Despite the noted catalytic activity of laccases towards phenolic groups of lignin, laccase cannot oxidize lignin completely. Consequently, mediators, acting as electron carriers, are needed to assist laccase in depolymerizing lignin and are considered a crucial factor in efficient and complete lignocellulosic residues degradation. In this study, the early degradation of cellulose and hemicellulose catalyzed by the cellulase and xylanase from *T. reesei* ZJ-09 could release various phenolic compounds which could act as natural mediators for laccase. In this way, the formed laccase–mediator systems (LMS) could expand the oxidation ability of laccase, which made the recombinant *T. reesei* have greater potential to effectively degrade lignocellulosic wastes as well as organic pollutants.

### Biodegradation of rice straw by *T. reesei*

**Fig. 4:**
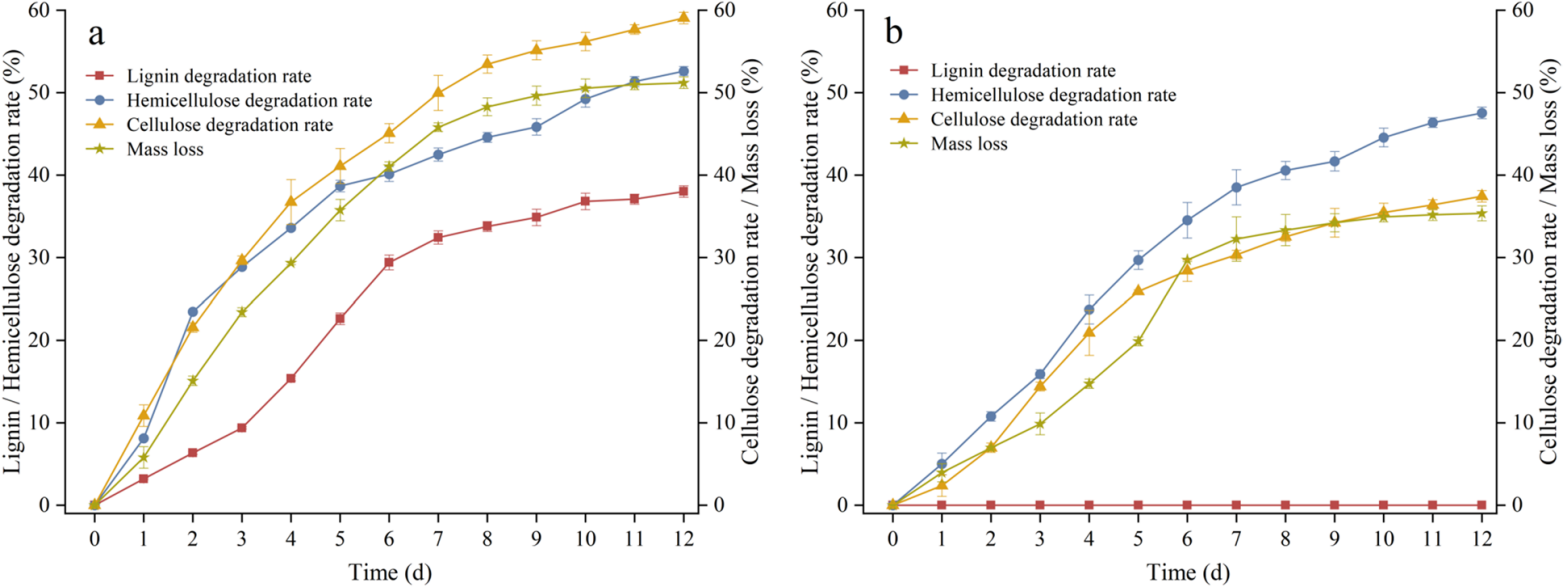
Degradation of rice straw by *T. reesei* ZJ-09 (**a**) and original strain (**b**) under optimized SSF conditions. Error bars represent the standard deviation of three independent repeats

Changes in the biomass compositions under SSF process are shown in Fig. 4. As it was expected, recombinant *T. reesei* ZJ-09 retained the strong cellulose and hemicellulose degradation ability of the original strain. The degrading ratio of cellulose and hemicellulose increased immediately after inoculation, reaching 21.41% and 23.64% on day 2, respectively, and then topped 59.12% and 52.61% on day 12, respectively.

Additionally, it is noteworthy that *T. reesei* ZJ-09 was able to degrade lignin effectively (Fig. 4a). Despite the lower removal rate during the initial phase of SSF, the lignin degradation ratio for *T. reesei* ZJ-09 had been growing with the laccase activity since day 4. The lignin degradation ratio was up to 38.05% on day 12. By comparison, the lignin degradation ratio of the original strain was undetectable.

The lower percentage of lignin makes cellulose and hemicellulose more available to the microorganism, which leads to higher biomass loss. As a result, the final mass loss of ZJ-09 was 1.4-fold higher to 51.16% compared to that of the original strain.

### POPs degradation by recombinant *T. reesei* koji

Target organic contaminants (nonylphenol, 2,4,5-trichlorophenol, and oxytetracycline) selected in this study are the three most recalcitrant and prevalent in the environment, which pose significant health risks to humans. The laccase-mediated removal rates of the three contaminants are lower than 15% when no mediator is added.

**Fig. 5:**
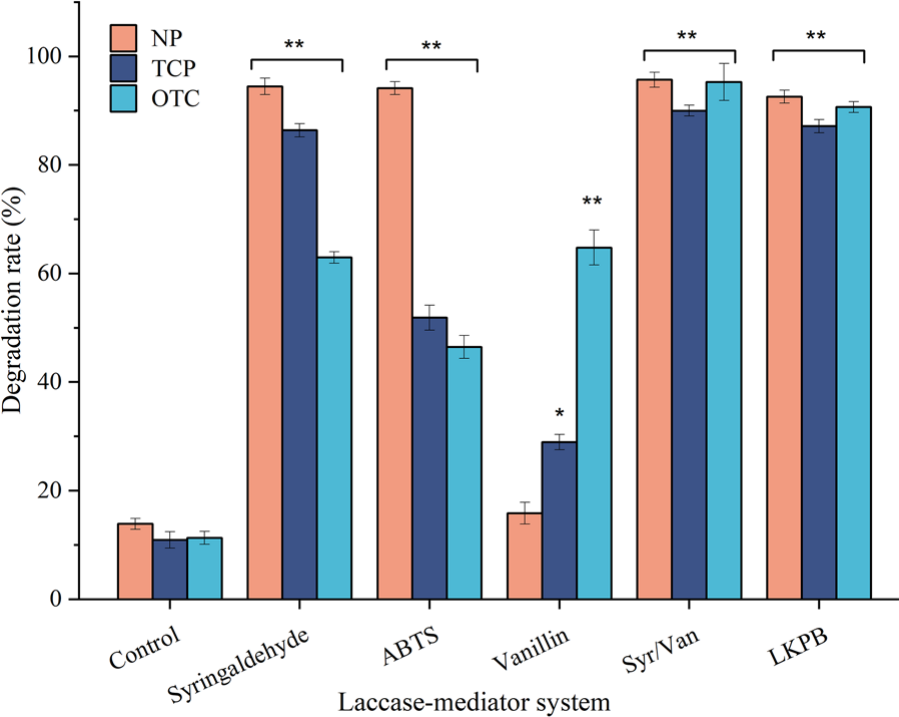
Degradation of POPs by different laccase–mediator systems. Individual POP on 50 mg/L was treated with 0.2 IU/mL laccase at pH 4.0, 50 ℃ for 4 h in the presence or absence of 0.3 mM individual mediator or Syr/Van complex with a ratio of 4:6. Control was performed on 50 mg/L individual POP with 0.2 IU/mL laccase without mediators. Residual POP concentrations were determined by HPLC. Error bars represent the standard deviation of three independent repeats. *p < 0.05, **p < 0.01 compared with control

As expected, the commonly used synthetic mediator ABTS could facilitate laccase-catalyzed degradation of inert chemicals. After reacting for 4 h, ABTS-mediated treatments achieved the removal rates of 94.28%, 52.01%, and 46.73% for nonylphenol (NP), 2,4,5-trichlorophenol (TCP), and oxytetracycline (OTC), respectively (Fig. 5). Regardless of the proven efficiency of the laccase/ABTS system, artificial mediators lead to additional costs, exhibit potential toxicity (Becker et al. 2016), and can cause laccase inactivation (Fillat et al. 2012). In comparison, natural mediators, considered as the true mediators for fungal laccases, are good alternatives to artificial ones given that they are more environmentally friendly and economically feasible.

As shown in Fig. 5, the removal rates were 94.55%, 86.42%, and 62.98% for NP, TCP, and OTC, respectively, by syringaldehyde/laccase system. Interestingly, an improved removal rate was observed when syringaldehyde and vanillin were present in combination, indicating the coexistence of various mediators could bring a synergistic effect in laccase–mediator systems. This was probably due to that not only the primary radical species, but also the secondary species produced by the chain reactions among primary radicals could act on the recalcitrant chemicals, resulting in the enhancement of the oxidation efficiency.

A similar result was found by POPs degradation by koji from SSF by recombinant *T. reesei* ZJ-09 (LKPB). Degradation reactions using supernatant from submerged fermentation of *T. reesei* ZJ-09 (control group) were also run along with the test. When no mediator was added, the 4 h removal rates mediated by LKPB (92.45% for NP, 87.21% for TCP, and 90.73% for OTC) were comparable to those achieved by syringaldehyde/vanillin co-mediator system, while those of the control group were all lower than 15%.

Previous studies showed that the combination of laccase and mediators can efficiently transform phenolic xenobiotics into less toxic compounds (Su et al. 2018). For example, the degradation of OTC by laccase–mediator system (LMS) showed a reduction in toxicity (Mir-Tutusaus et al. 2014) and antimicrobial activity (Yang et al. 2017). As for TCP, the toxicity, persistence, and biodegradability depend on the number and position of chlorine substituents in the phenolic ring (Rubilar et al. 2008). Çabuk et al. revealed that TCP was dechlorinated by laccase (Çabuk et al. 2012) and Liu et al. demonstrated that laccase could achieve a good detoxification effect while degrading chlorophenols (Liu et al. 2021). Also, the acute toxicity test pointed out that the detected metabolites of NP were less toxic than the parent compound after laccase treatment (Mtibaà et al. 2020).

However, the application of laccase is limited because it is hard to react with pollutants with high redox potentials alone (Su et al. 2018). It was found that natural phenolic compounds derived from plants can generate active free radicals and then act on inert chemicals as mediators for laccase, thus expanding the substrate spectrum of laccase. In this study, the degradation of rice straw catalyzed by enzyme cocktails secreted by *T. reesei* ZJ-09 during SSF could release various phenolic compounds, which then formed laccase–mediator system and expanded the oxidation ability of laccase.

In this way, the recombinant *T. reesei* have greater potential to effectively degrade lignocellulosic wastes and organic pollutants simultaneously without addition of mediators. The future application of LKPB in wastewater and soil remediation is worth exploring.

## Conclusions

Recombinant *T. *reesei ZJ-09 exhibited the ability of synchronous secretion of cellulase, xylanase, and laccase under SSF. Effects of key parameters on enzyme yield during the SSF process were investigated. Under the optimized SSF parameters, the FPA, xylanase activity and laccase activity reached 110.47 FPU/g, 5787.59 IU/g, and 24.45 IU/g, respectively, after 12 days of fermentation. When using rice straw as the carbon source, the resulting mass loss by recombinant *T. reesei* ZJ-09 was high up to 51.16%, which was significantly higher than that achieved by the original strain. The koji obtained from SSF of rice straw by recombinant *T. reesei* ZJ-09 effectively degraded recalcitrant organic pollutants (TCP, NP, and OTC) without the addition of mediators. The degradation rates were higher than those achieved by adding ABTS and vanillin as mediators and were comparable to those achieved by syringaldehyde and Syr/Van cocktail as mediators. The newly established recombinant *T. reesei* SSF system could be potential for economical and efficient lignocellulosic biomass conversion and bioremediation.

### Supplementary Information

**Additional file 1: Table S1.** β-glucosidase activity (BGA), cellobiohydrolase activity (CBHA), and endoglucanase activity (CMCase) from *T. reesei *under optimized SSF conditions using rice straw as substrate on day 12. **Table S2. **Compositions of lignocellulosic wastes used in this study.

## Data Availability

All data supporting this article’s conclusion are available.
