# Supplementary material for: Efficient biodegradation of straw and persistent organic pollutants by a novel strategy using recombinant Trichoderma reesei
Source: Bioresour Bioprocess. 2022 Aug 29;9(1):91. doi: 10.1186/s40643-022-00581-9 (PMC10992801; doi:10.1186/s40643-022-00581-9)
Supplement: Supplementary file 1 — Additional file 1: Table S1. β-glucosidase activity (BGA), cellobiohydrolase activity (CBHA), and endoglucanase activity (CMCase) from T. reesei under optimized SSF conditions using rice straw as substrate on day 12. Table S2. Compositions of lignocellulosic wastes used in this study. [file 40643_2022_581_MOESM1_ESM.docx]

**Additional file 1**

**Table S1** β-glucosidase activity (BGA), cellobiohydrolase activity (CBHA), and endoglucanase activity (CMCase) from *T. reesei* under optimized SSF conditions using rice straw as substrate on day 12

| **Strain** | **BGA (IU/g)** | **CBHA (U/g)** | **CMCase (IU/g)** |
| --- | --- | --- | --- |
| ZU-02 (control) | 15.35 ± 1.32 | 756.21 ± 9.32 | 1481.43 ± 10.24 |
| ZJ-09 | 13.78 ± 0.82 | 679.16 ± 7.92 | 1213.15 ± 9.16 |

Listed data represent mean values of triplicate samples.

**Table S2** Compositions of lignocellulosic wastes used in this study

| **Material** | **Cellulose (%)** | **Hemicellulose (%)** | **Lignin (%)** | **Ash (%)** |
| --- | --- | --- | --- | --- |
| CS | 41.3 ± 0.4 | 27.9 ± 0.5 | 20.2 ± 0.3 | 4.6 ± 0.4 |
| RS | 37.1 ± 0.6 | 23.4 ± 0.9 | 16.7 ± 0.5 | 2.6 ± 0.3 |
| WS | 29.5 ± 1.2 | 26.7 ± 1.0 | 19.8 ± 0.6 | 5.3 ± 0.6 |
| SCB | 39.4 ± 0.4 | 29.3 ± 0.8 | 22.4 ± 1.3 | 8.7 ± 0.5 |

Listed data represent mean values of triplicate samples.
